# Axolemmal nanoruptures arising from paranodal membrane injury induce secondary axon degeneration in murine Guillain‐Barré syndrome

**DOI:** 10.1111/jns.12532

**Published:** 2023-02-12

**Authors:** Madeleine E. Cunningham, Rhona McGonigal, Jennifer A. Barrie, Clare I. Campbell, Denggao Yao, Hugh J. Willison

**Affiliations:** ^1^ School of Infection & Immunity University of Glasgow Glasgow UK

**Keywords:** calpain, Guillain‐Barré syndrome, nanoruptures, secondary axon degeneration

## Abstract

The major determinant of poor outcome in Guillain‐Barré syndrome (GBS) is axonal degeneration. Pathways leading to primary axonal injury in the motor axonal variant are well established, whereas mechanisms of secondary axonal injury in acute inflammatory demyelinating polyneuropathy (AIDP) are unknown. We recently developed an autoantibody‐and complement‐mediated model of murine AIDP, in which prominent injury to glial membranes at the node of Ranvier results in severe disruption to paranodal components. Acutely, axonal integrity was maintained, but over time secondary axonal degeneration occurred. Herein, we describe the differential mechanisms underlying acute glial membrane injury and secondary axonal injury in this model. Ex vivo nerve‐muscle explants were injured for either acute or extended periods with an autoantibody‐and complement‐mediated injury to glial paranodal membranes. This model was used to test several possible mechanisms of axon degeneration including calpain activation, and to monitor live axonal calcium signalling. Glial calpains induced acute disruption of paranodal membrane proteins in the absence of discernible axonal injury. Over time, we observed progressive axonal degeneration which was markedly attenuated by axon‐specific calpain inhibition. Injury was unaffected by all other tested methods of protection. Trans‐axolemmal diffusion of fluorescent proteins  and live calcium imaging studies indirectly demonstrated the presence of nanoruptures in the axon membrane. This study outlines one mechanism by which secondary axonal degeneration arises in the AIDP variant of GBS where acute paranodal loop injury is prominent. The data also support the development of calpain inhibitors to attenuate both primary and secondary axonal degeneration in GBS.

## 1 INTRODUCTION

The primary targets of autoimmune injury in acute inflammatory demyelinating polyneuropathy (AIDP), the demyelinating form of Guillain‐Barré syndrome (GBS), are Schwann cell membranes. These comprise the internodal compacted myelin membranes, abaxonal membranes and the specialised non‐myelinated membranes of the nodal complex. Whilst the range of Schwann cell antigens in AIDP has yet to be fully defined, it is widely believed that complement fixing autoantibodies to glycolipids, including gangliosides, sulphated glycolipids and possibly other unidentified molecules, mediate the injury.^1^, ^2^, ^3^ Ultimately, Schwann cell injury results in conduction block due to nodal disorganisation, usually accompanied by segmental demyelination.^4^, ^5^, ^6^

The recovery rates in the acute motor axonal neuropathy (AMAN) variant of GBS and in AIDP are dependent upon the extent and site of axon degeneration.^7^, ^8^ In AMAN, where the axonal injury is the primary pathological event, recovery varies from very poor to complete, depending on the site and extent of axonal injury. In pure AIDP without axonal injury, segmental remyelination is an efficient process in peripheral nerve with variable and complete restoration of function.^9^, ^10^ When AIDP is complicated by secondary axon degeneration, this may result in permanent denervation, especially when the axon loss occurs proximally, and consequential long‐term functional loss.^3^, ^7^

The mechanisms by which axons undergo secondary axon degeneration in AIDP are unknown. Autopsy studies of AIDP patients have shown deposits of complement including the terminal membrane attack complex (MAC) over glial membranes,^11^ accompanied by infiltration of macrophages which phagocytose myelin debris.^12^ In AIDP patients with secondary axonal degeneration, studies have suggested a variety of causative mechanisms including compression of the axon due to infiltration of macrophage processes^13^ and rises in endoneurial fluid pressure at critical anatomical sites that might induce ischaemic and other injuries to axons.^14^ In rat models of experimental allergic neuritis (EAN) in which immune attack is targeted to myelin, degradation of the axon‐glial unit at nodes of Ranvier (NoR) with disruption of adhesion molecules and ion channels is a prominent feature,^4^ but the causal mechanisms linking these events to the concomitant axon degeneration have not been elucidated. In non‐inflammatory demyelinating neuropathies, notably Charcot‐Marie‐Tooth diseases, secondary axonal degeneration is widespread over a long timescale and attributed to energy failure, metabolic and neurotrophic factor deprivation.^15^ Many lines of evidence thus support the symbiotic interdependency between Schwann cells and axons that are essential for each other's survival and function, and that will vary considerably in different developmental and pathological contexts.

We recently reported the mouse model of Schwann cell membrane‐directed injury, in which anti‐GM1 antibody (Ab) plus complement targets the glial membrane, resulting in deposition of MAC pores at the distal paranodal glial membranes, and the disruption of nodal architecture.^5^ In the acute phase of this model, both ex vivo and in vivo, we observed the loss of many glial nodal complex markers, indicating paranodal disruption. Ultrastructural analysis also showed greatly swollen and distorted paranodal regions likely due to the influx of extracellular fluid and ions via MAC pores that activate calpain cleavage pathways, mechanistically similar to that occurring in our AMAN models.^16^, ^17^, ^18^, ^19^ At the acute timepoint in this glial model, axonal integrity remained intact; however, over time following more extended glial injury, both ex vivo and in vivo, secondary axonal degeneration developed. This experimental paradigm allows us to investigate potential mechanisms by which secondary axonal degeneration occurs following extended glial injury. Herein we describe one putative mechanism by which this secondary axonal degeneration occurs.

## 2 MATERIALS AND METHODS

### 2.1 Antibodies and reagents

Antibodies against glycolipids were generated and characterised as previously described.^20^, ^21^, ^22^ For studies targeting glial membranes in wild‐type mice, anti‐sulfatide antibody (GAME‐G3) was used. For studies where complex ganglioside expression was restricted to glial membranes, anti‐GM1 antibody (DG2) was used (Table 1).

**TABLE 1:** Genetically altered mice used in this study and anti‐glycolipid antibodies used for each genotype.

Mouse	Genetic alteration	Purpose	Original reference	Anti‐glycolipid antibody used
*GalNAc‐T* ^*−/−*^ *‐Tg(glial)* (Referred to as *Glial*)	GalNAc‐T expression driven by Plp promoter on GalNAc‐T^−/−^ background	Restriction of complex ganglioside expression to glial membrane	^32^	DG2 (anti‐GM1 IgG3)
B6.Cg‐Tg(Thy1‐CFP/S100B‐GFP)	GFP and/ or CFP expression driven by S100B and Thy1.2 respectively	Expression of CFP in neurons and axons and GFP in glial cells	^69^, ^70^	GAME‐G3 (anti‐sulfatide IgG3)
*hCAST*	Human calpastatin Expression driven by Prp promoter	Overexpression of human calpastatin in neurons and axons	^71^	GAME‐G3 (anti‐sulfatide IgG3)
*SARM1* KO	Knock out of SARM1	*SARM1* KO mice show delayed Wallerian degeneration of axons	^72^	GAME‐G3 (anti‐sulfatide IgG3)
*Thy1‐*TNXXL	TNXXL Expression driven by Thy1.2 promoter	Expression of genetically encoded calcium indicator TNXXL in axons	^54^	GAME‐G3 (anti‐sulfatide IgG3)
	*Crosses*			
*hCAST* × G*lial*	Cross of *hCAST* and *GalNAc‐T* ^*−/−*^ *‐Tg(glial)* lines	Axonal Calpastatin overexpression on *GalNAc‐T* ^*−/−*^ *‐Tg(glial)* background	n/a	DG2 (anti‐GM1 IgG)

Abbreviations: hCAST, human calpastatin; SARM1, sterile alpha and armadillo motif‐containing protein 1.

The following commercial antibodies were used: rat anti‐myelin basic protein (MBP; Bio‐Rad Cat# MCA409S, RRID:AB_325004; 1/500), mouse anti‐phosphorylated neurofilament‐H (NF‐H) antibody SMI‐31 (BioLegend Cat# 801602, RRID:AB_2715851; 1/1500), mouse anti‐MAC (Agilent Cat# M0777, RRID:AB_2067162; 1/50), mouse anti‐AnkyrinB (UC Davis/NIH NeuroMab Facility, N105/17; RRID: RRID:AB_10673094, 1:300), rabbit anti‐pan‐Neurofascin (gifted from Peter Brophy, University of Edinburgh, Edinburgh, UK; 1:1000); rabbit anti‐green fluorescent protein (GFP; abcam Cat# ab6556, RRID:AB_305564; 1/500).

Secondary antibodies were prepared at a 1/500 dilution in PBS with 3% normal goat serum (NGS): Alexa Fluor 555 conjugated anti‐rat IgG (Thermo Fisher Scientific Cat# A‐21434, RRID:AB_2535855), Alexa Fluor 647 conjugated anti‐mouse IgG1 (Thermo Fisher Scientific Cat# A‐21240, RRID:AB_2535809), Alexa Fluor 488 and 555 conjugated anti‐mouse IgG2a (Thermo Fisher Scientific Cat# A‐21131, RRID:AB_2535771 and Cat# A‐21137, RRID: AB_2535776, respectively), Alexa Fluor 488 conjugated anti‐mouse IgG3 (Thermo Fisher Scientific Cat# A‐21151, RRID:AB_2535784) and Alexa Fluor 488 and 647 conjugated anti‐rabbit IgG (Cat# A‐11008, RRID:AB_143165 and Cat# A27040, RRID:AB_2536101, respectively).

Ringer's solution was prepared to a final concentration of 116 mM NaCl, 4.5 mM KCl, 23 mM NaHCO_3_, 1 mM NaH_2_PO_4_, 11 mM glucose, 1 mM MgCl_2_ and 2 mM CaCl_2_. For the addition of exogenous calpain inhibitor, AK295 was made up in dimethyl sulfoxide (DMSO) and added to Ringer's at 100 μM. For studies using lactate, L‐lactate (Sigma‐Aldrich, Cat# L7022) was made to a 1 M stock in dH_2_O and added to the Ringers at 50 μM. For reactive oxygen species (ROS) inhibition, the ROS inhibitor YCG063 (Merck Cat# 330997‐95‐0) was diluted to a stock concentration in DMSO as per manufacturer's instructions and used at 100 μM in Ringer's. For dextran dye studies, fixable 3 or 70 kDa dextran conjugated to tetramethylrhodamine (Thermo Fisher Scientific Cat# D3308 and D1818 respectively) were used at a concentration of 1 mg/mL.

### 2.2 Mice

Studies were carried out in accordance with the U.K. Animals (Scientific Procedures) Act, 1986. Wild‐type mice used in these studies were C57 Bl/6 mice. All genetically altered mice used for acute and extended ex vivo glial injury paradigms are described in Table 1 and all are on a C57BL/6 background. Mice used were both male and female sexes and aged between 4 and 10 weeks.

### 2.3 Experimental design and statistics

Sample size for ex vivo models was estimated based on a priori power calculations from pilot studies with G*power analysis software (v.3.1.9.4). For studies using anti‐GM1 antibody, an effect size of 1.78, an alpha error probability of 0.05 and a power of 0.8 was used to calculate group sizes. For studies using GAME‐G3 antibody, an effect size of 3.51, an alpha error probability of 0.05 and a power of 0.8 was used to calculate group sizes.

Immunofluorescent images were analysed blinded by a single observer for each complete experimental dataset. All data are displayed as mean ± SE of the mean (SEM) and relevant statistical tests and mouse numbers used for each analysis are described in the figure legends. Statistics were performed using GraphPad Prism (version 6.07), as was the generation of all graphs

### 2.4 Ex vivo injury models

Triangularis sterni nerve‐muscle explants from wild‐type, *GalNAc‐T* ^*−/−*^ *‐Tg(glial)* (referred to hereafter as *Glial)*, *SARM1* KO or *hCAST* × *Glial* mice were dissected and maintained in oxygenated Ringer's solution. Acute and extended glial injury was performed as described previously.^5^ Briefly, explants were incubated with either anti‐sulfatide antibody or anti‐GM1 antibody at 100 μg/mL alone (control) or with 40% normal human serum (NHS) as a source of complement (injury) in Ringer's. The antibody used was dependent on the ganglioside expression in the mice; *Glial* and *hCAST* × *Glial* only express complex gangliosides on glial cells and therefore anti‐GM1 antibody DG2 could be used to induce glial injury. In all other genotypes, gangliosides are expressed ubiquitously, therefore the anti‐sulfatide antibody GAME‐G3 was used to target injury to the glial membrane (Table 1). Antibodies to both GM1 and sulfatide are clinically relevant antibodies in AIDP patients, with varying proportions of patients being seropositive for these antibodies.^2^, ^3^, ^23^, ^24^

For acute injury, explants were incubated in control or injury solution for 4 h at 32°C. Following injury with antibody and complement, explants were washed 3× with Ringer's then fixed for 20 min in 4% paraformaldehyde (PFA) at 4°C, followed by 5‐min washes in PBS, 0.1 M glycine and PBS. For some studies, exogenous calpain inhibitor AK295^25^ was added along with the injury solution at a concentration of 100 μM.

For extended injury, explants were incubated for 20 h at room temperature (RT) then washed and fixed as above. To decipher potential mechanisms of secondary axon degeneration, extended injury paradigms were either carried out in transgenic mice (*SARM1* KO or *hCAST* × *Glial* mice) or with a treatment added to wild‐type explants during their overnight incubation (50 μM lactate or 100 μM of ROS inhibitor YCG063).

For dye application studies, extended glial injury was performed as described above, then tissue was washed, and, prior to fixation, dextran dyes were added at 1 mg/mL for 2 h at 37°C, followed by 4× 30 min washed with Ca^2+^ free Ringer's solution to reduce membrane activity. Explants were then fixed as described above.

Following fixation and washes, explants were incubated for 10 min in freezing ethanol at −20°C then primary antibodies were added overnight in PBS containing 3% NGS and 0.5% Triton X‐100. The following day, subtype‐specific secondary antibodies were added for 2 h at RT then explants were washed and mounted on APES‐coated slides in Citifluor antifadent mounting medium (Citifluor, USA).

### 2.5 Immunofluorescent image capture

Fluorescent images were captured using a Zeiss AxioImager Z1. For illustrative images, an Apotome attachment was used.

### 2.6 Live calcium imaging studies

Nerve‐muscle explants were maintained in fresh Ringer's solution. Baseline images were taken (described below) then explants were incubated with monoclonal anti‐sulfatide antibody GAME‐G3 (100 μg/mL) along with 40% NHS in Ringer's for 4 h at RT. Explants were washed 3× with Ringer's solution before every subsequent image was taken and returned to Ab/NHS solution in between images.

Images were taken up to 10 h post‐addition of antibody and NHS. Explants were then fixed as described above and TS muscles were removed and frozen down at −80°C. Images were captured as previously described.^18^ Briefly, Z‐stacks of explant regions replete with neuromuscular junctions (ranges included in figure legends) were acquired using a Zeiss LSM7 MP system, with a 20×/1.0NA water‐immersion objective lens and a tuneable titanium/sapphire solid‐state 2‐photon excitation source (Chameleon Ultra II; Coherent Laser Group), tuned to 840 nm. Light was first filtered through a 685‐nm LP dichroic. For FRET imaging, an LSM binary gallium arsenide phosphide (GaAsP) photodetector module was used with a CFP/YFP filterset cube (BP 455‐500/BP 525‐570) with an LP510 dichroic mirror. To minimise imaging time and prevent photodamage to the tissue, images were taken with 1.2 μm z‐slice with 1× line averaging.

### 2.7 Image analysis and quantification

Average intensity of GFP was measured along a 10 μm section spanning the final distal NoR determined by a gap in MBP staining. This was achieved using the line feature in the Fiji distribution of ImageJ software (version 1.52b).^26^

For presence and absence of other immunofluorescent markers or dyes, images were analysed blinded by a single investigator. For pan‐Neurofascin staining, two neurofascins have distinct staining patterns allowing their discrimination. Here we quantified two outputs: (1) Immunostaining that appeared normal and consisted of both NF155 and NF186 (if only nodal NF186 was present, these were counted as absent); (2) Length of immunostaining present. If staining was completely absent no measurements were made.

Axonal calcium presence was measured by calculating ratios of citrine/CFP in *Thy1*‐TNXXL mice as previously described.^18^ The morphology of axons was classed as either “normal”, “swollen” or “fragmented” at each timepoint measured. Fragmented axons were those displaying separation between segments of the axon even after the image stack was contrast adjusted. Calcium FRET images are displayed as ratiometric images which were created as previously described.^18^

## 3 RESULTS

### 3.1 Terminal complement pores disrupt glial membrane integrity

**FIGURE 1:**
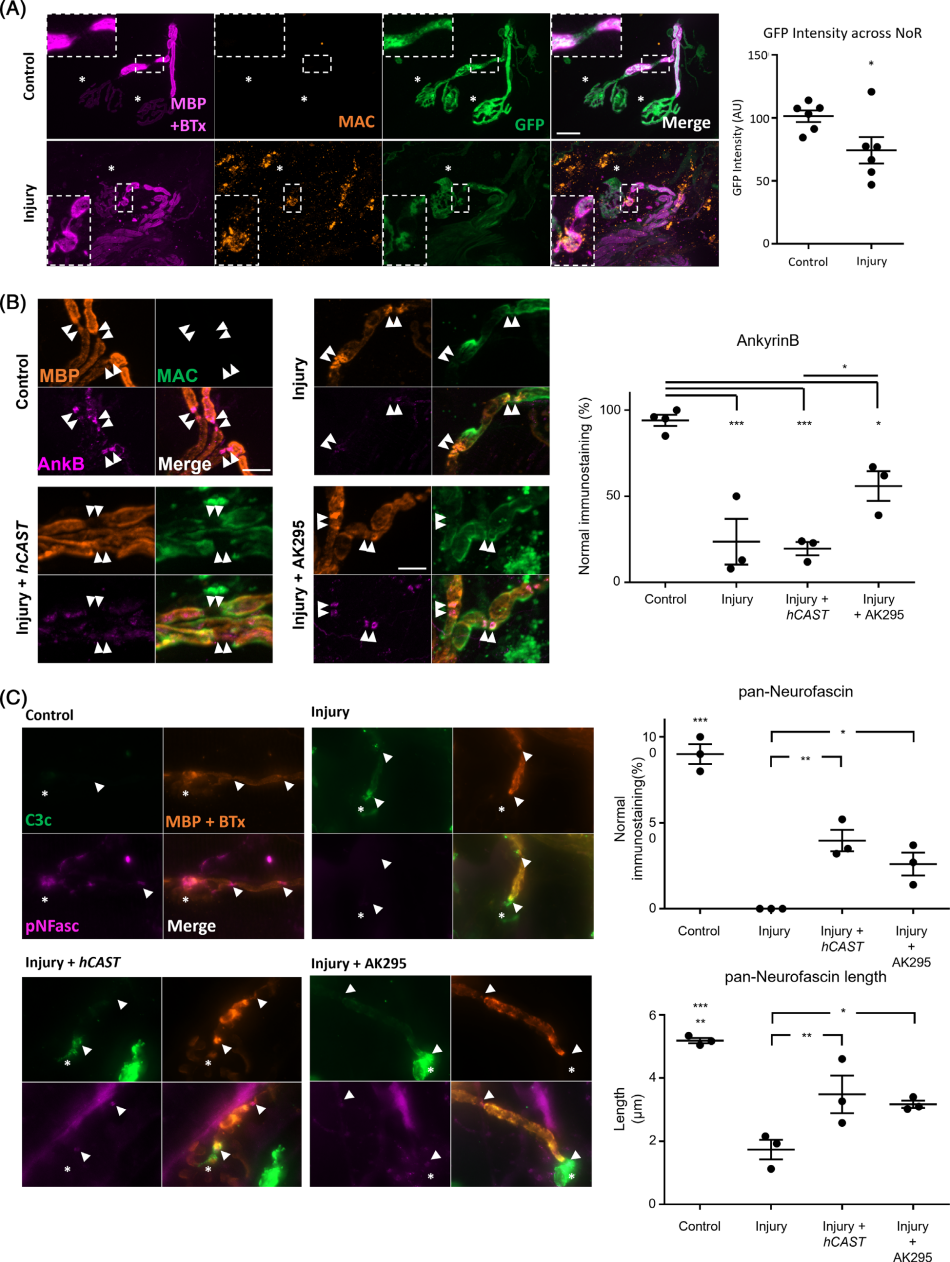
Deposition of MAC causes glial membrane disturbance leading to disruption of glial cytoskeletal and cell adhesion molecules by glial calpains. (A) Triangularis sterni nerve‐muscle preparations from B6.Cg‐Tg(Thy1‐CFP/S100B‐GFP) mice, which express green fluorescent protein (GFP) in Schwann cell cytoplasm were treated ex vivo acutely (4 h) with anti‐sulfatide antibody alone (Control) or with a source of complement (Injury). Deposition of membrane attack complex (MAC) at glial paranodal regions was accompanied by a reduction in the intensity of GFP measured across the final node of Ranvier (NoR). Insets represent magnified region from boxed area. BTx, α‐bungarotoxin, asterisk represents site of neuromuscular junction; MBP, myelin basic protein. Scale bar = 20 μm. Results are represented as the mean ± SEM. n = 6/treatment; on average 29 NoR/mouse were analysed (range 19‐51). Data were compared by a paired two‐tailed *t*‐test, * signifies *P* < 0.05. (B) Nerve‐muscle preparations from *Glial* or *hCAST* × *Glial* mice were treated acutely ex vivo with anti‐GM1 Ab alone (Control) or with a source of complement (Injury) in the presence or absence of exogenous calpain inhibitor AK295. Loss of nodal protein (magenta) immunostaining at the NoR due to injury was assessed; the site of expected staining is indicated by arrowheads. Representative images demonstrate nodal protein localisation coinciding with complement deposition (green). AnkyrinB (AnkB) immunostaining was significantly reduced compared to control for each treatment. A significant protection was conferred by AK295. (C) A pan‐neurofascin (pNfasc) antibody was used to assess paranodal NF155 and nodal NF186. NoR with normal paranodal pNFasc immunostaining significantly decreased following *Glial* injury and was partially restored by both *hCAST* expression and AK295. Scale bars = 5 μm. Results are represented as the mean ± SEM. n = 3/treatment; on average 24 NoR/mouse were analysed (ranges 12‐44 AnkB, 16‐34 pNFasc). One‐way ANOVA, followed by Tukey post‐hoc tests were used. *** signifies *P* < 0.001, ** signifies *P* < 0.01 and * signifies *P* < 0.05.

Following acute immune‐mediated injury to the glial membranes, we show deposits of MAC at the distal NoR, particularly at paranodal regions (Figure 1A). We have previously shown that CFP expressed in axonal cytoplasm is lost through MAC pores when axons are directly targeted with anti‐ganglioside antibody and a source of complement.^27^, ^28^ Using mice that express cytosolic GFP driven by the S100B promoter, and therefore present in Schwann cell cytoplasm, we here demonstrate that following acute (4 h) glial injury, deposits of MAC on the paranodal glial membrane are accompanied by a significant decrease in GFP fluorescent intensity, indicating a loss of GFP likely through the complement pores (Figure 1A, two‐tailed paired *t* test, *P* < 0.05). As previously observed, MBP disruption was often noted in this model^5^ as was S100 staining of Schwann cells (data not shown).

### 3.2 Glial calpains disrupt glial cytoskeletal and cell adhesion molecules

To determine the role of calpain in the disruption of the paranode following acute immune‐mediated injury to the glial membrane, we used genetic and exogenous calpain inhibition strategies and studied the structural and cell adhesion paranodal proteins AnkyrinB and NF155, respectively. As shown previously, AnkyrinB immunostaining is disrupted when glial membranes are targeted compared to control.^5^ There is a significant reduction in AnkyrinB in all injured tissue compared to control (Figure 1B, one‐way ANOVA F[2,7] = 50.9, *P* < 0.001, Tukey's post‐hoc multiple comparison test). However, this disruption is partially attenuated by glial calpain inhibition, as shown by AnkyrinB protection by global application of AK295, but not with axonal expression of the endogenous calpain inhibitor human calpastatin (*hCAST)* (Table 1). Whilst protection using AK295 is significant compared to *hCAST*, immunostaining patterns remain significantly impaired compared to control tissues. These results indicate that glial calpain influences AnkyrinB disruption, but other factors are also contributing to paranodal disruption.

We next examined a pan‐Neurofascin antibody that binds both glial NF155 on the paranodal loop membranes, and axonal NF186 at the nodal axolemma, to help determine calpain involvement in paranodal disruption (Figure 1C, one‐way ANOVA F[3,8] = 49.1, *P* < 0.001, Tukey post‐hoc multiple comparison test). As shown before, NoR with intact pan‐Neurofascin immunostaining for both isoforms significantly decreased when the glial membrane was injured compared to control. When present, immunostaining was limited to the nodal gap, and therefore presumed to be NF186 and considered pan‐neurofascin‐negative in quantitative analysis. Both axonal *hCAST* expression and global AK295 application provided comparable and modest protection compared to injury conditions without any calpain inhibition. However, the protection appeared incomplete and the immunostained domain appeared shorter. Therefore, we next measured the total length of staining present across the NoR (one‐way ANOVA F[3,8] = 17.01, *P* < 0.001, Tukey's post‐hoc multiple comparison test). There was a significant reduction in total pan‐neurofascin length in all injured tissue compared to control. Again, there was a comparable but incomplete improvement compared to injury in the presence of calpain inhibition by both *hCAST* expression and application of AK295.

### 3.3 Axonal calpain inhibition attenuates secondary loss following glial injury

**FIGURE 2:**
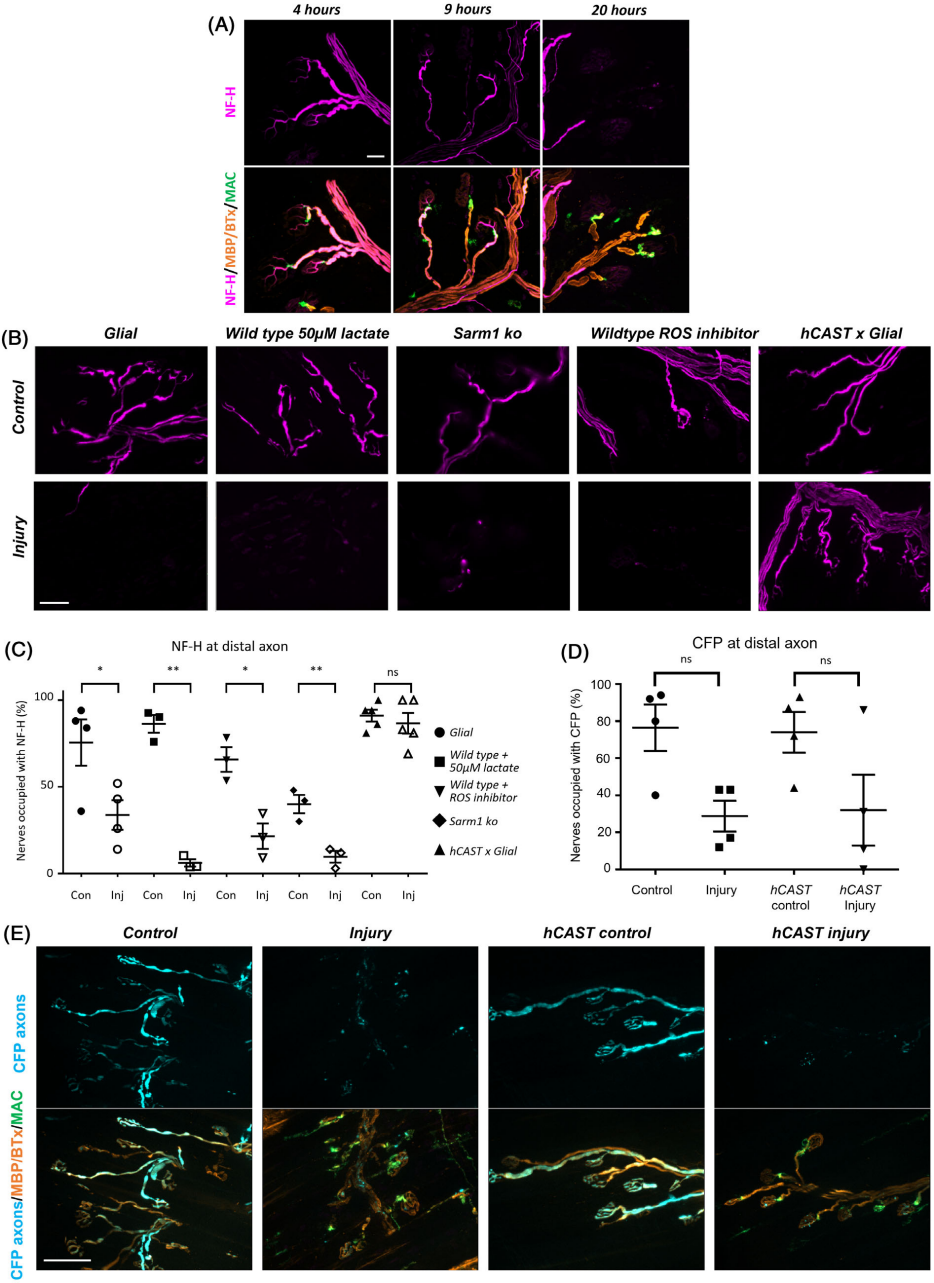
Axonal calpain drives secondary axon loss following extended glial injury. (A) Triangularis sterni nerve‐muscle explants from wild‐type mice were treated ex vivo with anti‐sulfatide Ab and a source of complement. After acute injury (4 h), neurofilament heavy (NF‐H) staining remained intact. Subsequently, progressive loss of NF‐H staining occurred over time. Scale bar = 50 μm. (B) Explants following extended ex vivo injury (20 h) with anti‐GM1 Ab (*Glial, hCAST* × *Glial*) or anti‐sulfatide Ab (wild‐type, *SARM1* KO) either alone (Control, Con) or with a source of complement (Injury, Inj) under modified conditions assessing metabolism (50 μm lactate, wild‐type), reactive oxygen species (ROS) (ROS inhibition, wildtype), the SARM1 pathway (*SARM1* KO mice), or calpain inhibition (*hCAST* × *Glial*). Loss of NF‐H (magenta) immunostaining or endogenous cytoplasmic cyan fluorescent protein (CFP) along the distal axon was assessed following injury. Illustrative images demonstrate the loss of NF‐H immunostaining in all injured groups with the exception of *hCAST* × *Glial*. (C) NF‐H immunostaining occupancy in distal axons was significantly reduced compared to control and not improved in the presence of 50 μm lactate, *SARM1 ko* or with ROS inhibition. However, a significant protection was conferred by calpain inhibition in *hCAST* × *Glial* tissue. (D) CFP loss occurred in *Glial* injured tissue compared to control and was not protected by *hCAST* expression. (E) Illustrative images depicting CFP loss in *hCAST* × *Glial* injured tissue. Scale bars = 50 μm. Results are represented as the mean ± SEM. n = 3‐5/treatment; on average 44 distal nerves/mouse were analysed (33‐48 *Glial*, 24‐54 lactate, 36‐72 *SARM1* ko, 11‐38 ROS inhibitor, 14‐82 *hCAST* × *Glial*, 24‐82 CFP). Student's t‐test and one‐way ANOVA, followed by Tukey's post‐hoc tests were used to assess significant differences. *** signifies *P* < 0.001, ** signifies *P* < 0.01 and * signifies *P* < 0.05.

At the acute timepoints described above, axonal protein NF‐H immunostaining was previously shown to be unaffected.^5^ Here, we show again that NF‐H is intact following acute glial injury, but that NF‐H is progressively lost over extended periods of injury, first undergoing fragmentation and then disappearing (Figure 2A). We next assessed the various mechanisms that might underlie this secondary NF‐H immunostaining loss following extended glial injury ex vivo, using a range of biochemical mediators and transgenic mice (Figure 2B,C, pairings were compared by individual one‐tailed, paired student's t‐tests). To study axonal metabolic deficiency, we applied 50 μM lactate as a source of energy to our preparations. There was no improvement in NF‐H immunostaining following this treatment (*P* = 0.0041 vs. control). The sterile alpha and armadillo motif‐containing protein 1 (SARM1) pathway have a role in axon degeneration following nerve transection,^29^ which can be attenuated in *SARM1* ko mice (Table 1). However, in *SARM1* ko mice explants, NF‐H immunostaining loss was not prevented when our injury paradigm was applied (*P* < 0.0022 vs. control). Considering that the glial membrane could potentially release cytotoxic mediators, we incubated tissue in the presence of ROS inhibitors. We observed no benefit of ROS inhibition on secondary loss of axon integrity in our model (*P* = 0.0364 vs. control). We considered that axonal calpain might be playing a key role. Using transgenic *hCAST* mice, we show that axonal calpain inhibition attenuates NF‐H immunostaining loss following glial injury (*P* = 0.2120 vs. control). Significantly, whilst NF‐H immunostaining is protected by *hCAST* expression, the cytoplasmic CFP loss is not attenuated (Figure 2D,E, one‐way ANOVA, F[3,12] = 3.792, *P* < 0.05), which suggests CFP leakage into the extracellular environment through a disrupted axonal membrane.

### 3.4 Secondary axon injury is caused by nanopore formation in axon membrane

**FIGURE 3:**
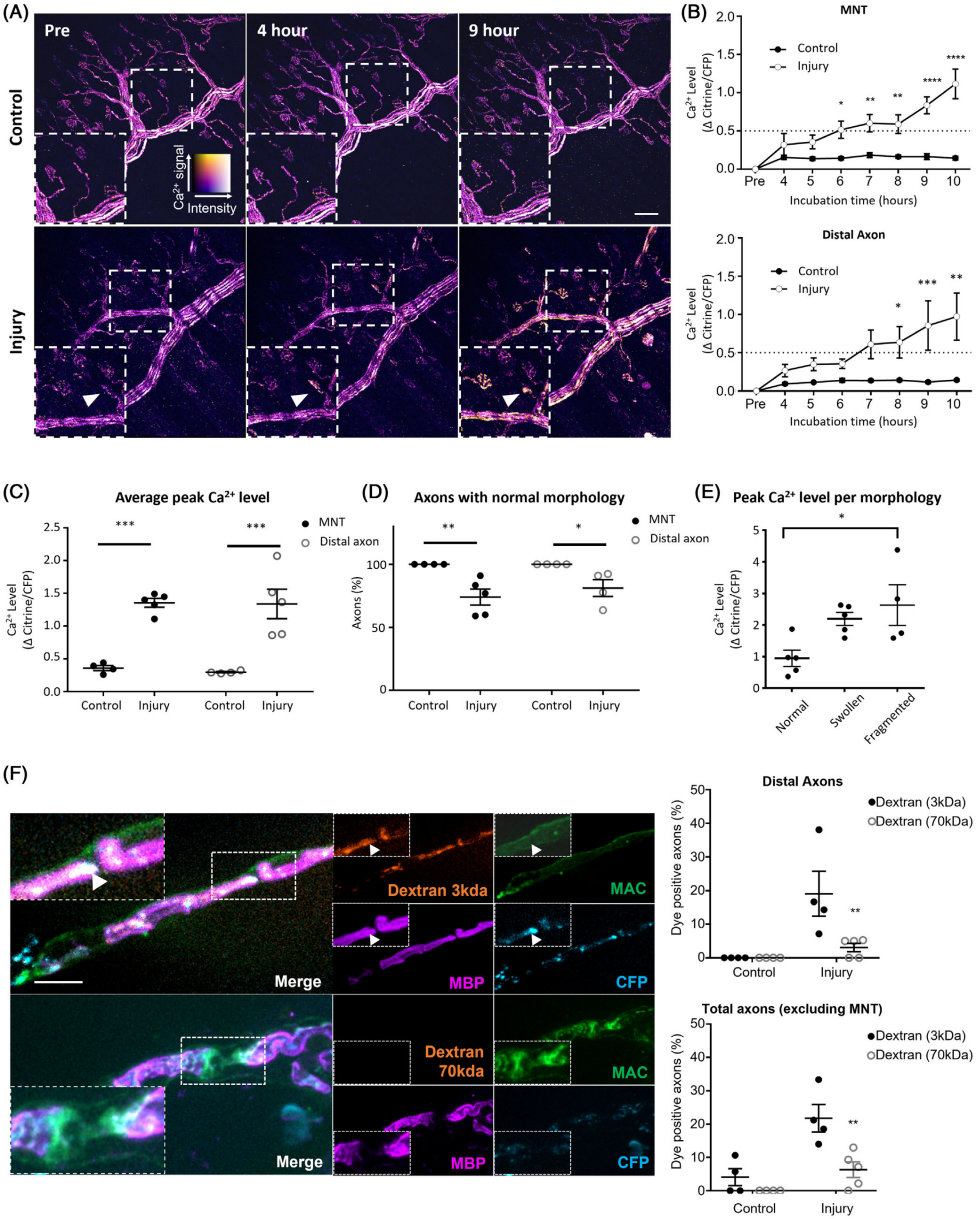
Following extended glial injury, pores form in axonal membranes allowing influx of calcium with pathological consequences. Triangularis sterni nerve‐muscle explants from *Thy1*‐TNXXL mice were imaged at baseline (Pre) then treated ex vivo for 4 h with anti‐sulfatide antibody (Ab) alone (Control) or with a source of complement (Injury). Subsequent images were captured hourly up to 10 h. (A) Ratiometric calcium images showing axonal calcium signals. Colour key demonstrates relative calcium levels. Scale bar = 50 μm. (B) Calcium rose significantly compared to controls at motor nerve terminals (MNTs; arrows) after 6 h and at distal axons at 8 h. (C) Average peak calcium levels for each mouse showed that injured mice had significantly higher axonal calcium than control counterparts at both MNTs and distal axons. (D) normal morphology was reduced at both measured sites in injured explants. n = 4 control, n = 5 injury; on average 15 axons were measured per mouse (range 11‐21). Two‐way ANOVA followed by Sidak's post‐hoc tests were used to assess significant differences. ****signifies *P* < 0.0001, ***signifies *P* < 0.001, **signifies *P* < 0.01 and *signifies *P* < 0.05. (E) Peak levels of calcium were significantly higher in “fragmented” axons by the conclusion of the experiment than those whose morphology remained normal. n = 5 for normal and swollen, n = 4 fragmented. One‐way ANOVA with Tukey's post‐hoc test was used to assess significant differences. *signifies *P* < 0.05. (F) Explants from B6.Cg‐Tg(Thy1‐CFP/S100B‐GFP) with single fluorescence for axonal cyan fluorescent protein (CFP) were incubated with either anti‐sulfatide Ab alone (Control) or with a source of complement (Injury) for 20 h. Explants were then incubated with different‐sized dextrans for 2 h. No differences were observed between dextrans in control tissues. In Injured tissues, significantly less penetration was seen with 70 kDa dextran than with 3 kDa dextran. Illustrative images are shown of injured tissue with differently sized dextrans. White arrowheads show 3 kDa Dextran colocalising with remaining axonal CFP. 3 kDa Dextran also does not closely associate with areas of MAC or MBP positive staining. n = 3 3 kDa, n = 4 70 kDa; on average 22 single axons/mouse (range 16‐33) and 40 total axons (range 20‐54) were analysed per mouse. Two‐way ANOVA followed by Sidak's post‐hoc tests were used to assess significant differences. **signifies *P* < 0.01. All results are represented as the mean ± SEM. Scale bar = 10 μm.

As shown above, inhibition of calpain protects axons from the loss of NF‐H immunostaining, indicating the involvement of elevated intra‐axonal calcium levels in secondary axonal damage following glial injury. To investigate this directly, we used *Thy1*‐TNXXL mice which express the genetically encoded calcium indicator TNXXL^30^ only in their axons to look at axonal‐specific changes in calcium in our glial injury model. As with the extended glial injury model, explants were maintained at RT during incubation with Ab and NHS. Control explants maintained stable axonal calcium levels throughout. In injured explants, a small number of motor nerve terminals (MNT) began to show alterations in calcium at early timepoints, becoming increasingly frequent at later time points (Figure 3A). We compared Ca^2+^ levels (measured by the change in ratios of citrine/CFP) over time at MNTs (Figure 3B, two‐way ANOVA for treatment F(1,45) = 97.30, *P* < 0.0001, for time F(7,45) = 10.05, *P* < 0.0001, interaction F(7,45) = 6.130) and distal axons (Figure 3B, two‐way ANOVA for treatment, F(1,45) = 38.82, *P* < 0.0001; for time F(7,45) = 3.942, *P* < 0.01; interaction F(7,45) = 2.499). At the MNT, Ca^2+^ levels became significantly higher than controls at 6 h with largest rises occurring at 9 and 10 h (Sidak's post‐hoc multiple comparisons test). Distal axons innervating MNTs followed the pattern of MNT Ca^2+^ flux closely. When the average peak Ca^2+^ level recorded for each axon was compared between control and injured explants, significantly higher Ca^2+^ was present at both MNT and distal axons (Figure 3C, two‐way ANOVA for treatment, F(1, 14) = 57.72, *P* < 0.0001; for axon type measured F(1, 14) = 0.0880, *P* = 0.7710; interaction F(1, 14) = 0.0291, Sidak's post‐hoc multiple comparisons test). Injured MNTs and distal axons also had a lower propensity for normal morphology (Figure 3D, two‐way ANOVA for treatment, F(1, 13) = 20.52, *P* = 0.0006; for axon type measured F(1, 13) = 0.5166, *P* = 0.4850; interaction F(1, 13) = 0.5166, Sidak's post‐hoc multiple comparisons test). Fragmented axons had a higher peak Ca^2+^ level than normal axons, indicating the involvement of Ca^2+^ as an associated, if not causal, factor in axonal damage (Figure 3E, one‐way ANOVA F[2, 11] = 5.485, *P* = 0.0223, Tukey's post‐hoc multiple comparison test).

As the loss of intra‐axonal cytoplasmic CFP is not prevented, even in the presence of axonal calpain inhibition, we considered that nanoruptures in the axolemmal membrane might exist, through which CFP was leaking into the extracellular milieu. This would also be consistent with calcium influx through axolemmal membrane pores, as opposed to the release of calcium from intracellular stores. We sought to confirm this and determine the relative size of the axonal membrane pores by using different molecular weight dextran dyes following extended glial injury. We analysed the presence of dyes at distal axons (Figure 3F, two‐way ANOVA, for treatment (control vs. injury), F(1,13) = 11.85 *P* < 0.01; for dye F(1,13) = 6.208 *P* < 0.05; interaction F(1,13) = 6.208) and also in all axons within the field of view (FOV), excluding MNTs (two‐way ANOVA, for treatment (control v injury), F (1, 13) = 19.96 *P* < 0.001; for dye F (1, 13) = 13.23 *P* < 0.01; interaction F(1,13) = 4.474). MNTs were excluded from analysis as they often showed the presence of dyes in control tissue, presumably as this specialised membrane is constitutively active with endocytic uptake. Post‐hoc analysis using Sidak's multiple comparisons test revealed a significantly higher presence of the smaller molecular weight dye (3 kDa) than the larger (70 kDa) dye both when distal axons were measured alone and when all axons within the FOV were measured (Figure 3F, both *P* < 0.01).

## 4 DISCUSSION

The mechanistic distinctions underlying primary and secondary axonal injury seen in demyelinating and axonal variants of GBS are poorly understood, owing to the lack of suitable animal models that unambiguously distinguish the two clinical variants. In particular, the prominent glycolipid target for auto‐antibodies, GM1 ganglioside, along with other ganglioside targets is expressed in both Schwann cells and neurons. Consequently, anti‐GM1 antibodies may simultaneously target and injure both Schwann cell (myelin and paranodal) and axonal membranes, making it practically impossible to disentangle primary and secondary axonal injury pathways.^6^, ^31^

To circumvent this impasse, we previously generated glycosyltransferase transgenic mice to specifically express GM1 and other complex gangliosides in either axonal or glial membranes.^32^ This allows us to create mutually distinct models of AMAN and AIDP mediated by a single anti‐GM1 antibody with a source of complement.^5^ To independently validate the AIDP model, we reproduced the salient findings of the transgenic mouse model with a recently developed anti‐sulfatide monoclonal antibody that binds avidly to and injures the Schwann cell paranodal membrane in wild‐type mice that have a normal composition of both glial and axonal gangliosides. In previous work, we used these AIDP model systems to catalogue the concurrent disruption of both glial and axonal paranodal and nodal proteins, including Ankyrins B and G, NF‐155/186, gliomedin, Caspr and Nav1.6. We speculated these changes were due to calcium‐activated, calpain‐mediated paranodal loop distortion leading to an indirect effect on axonal binding partners and axonal integrity. In this study, we investigate the mechanisms leading to axonal injury following subacute glial injury.

### 4.1 Acute injury directed to the glial paranode results in GFP loss through MAC pores with calpain‐mediated disruption

MAC pores allow uncontrolled bidirectional flow of solutes through membranes. In mice expressing soluble GFP in the glial cytoplasm, acute glial injury due to MAC pore deposition results in a decrease in the intensity of cytoplasmic GFP. The most plausible mechanism is that fluorescent protein leaks from the injured membrane through the MAC pores as we previously observed for MAC pores in the axonal membrane of axonally expressing CFP mice.^16^, ^27^, ^28^ Extracellular calcium is likely also concomitantly entering paranodal loops through MAC pores. These results align with our previous findings demonstrating ultrastructural consequences of this injury in an in vivo setting, showing swellings and morphological disturbance of the paranodal loops.^5^

### 4.2 Acute glial injury is partially mediated by glial, but not axonal calpains

To demonstrate the role of calpain 1 and 2 we used two strategies for calpain inhibition: (a) the exogenous calpain inhibitor AK295 which can freely diffuse through all membranes in the tissue, thus inhibiting both glial and axonal calpain; and (b) *hCAST* mice which express the endogenous calpain inhibitor calpastatin selectively in their neurons and axons. We have recently demonstrated that the expression of *hCAST* in axons is sufficient to protect axons from structural damage in a model of primary axonal injury. Here, we show that, axonal calpain inhibition has no glial protective effect acutely, whereas both glial and axonal protection is seen with AK295. The partial protection seen with AK295 is likely due to incomplete calpain inhibition resulting from insufficient tissue penetration or be concentration dependent. Immunohistological readouts may also be affected by tissue swelling and molecular distortion.

Pan‐Neurofascin staining was moderately protected by both axonal calpain inhibition in *hCAST* mice, and by exogenous AK295. The localisation of pan‐Neurofascin staining correlates with the paranodal region and, therefore, assumed to be NF155, was absent in injury. Consequently, it seems that NF155 is disrupted by glial membrane injury and that this is in part mediated by calpains. NF155 is partially tethered by AnkyrinB at the glial paranodal membrane^33^; however, during development there may be redundancy in the requirements for this interaction.^34^ Nevertheless, in our severe acute injury model, loss of AnkyrinB by calpain cleavage and membrane distortion does also appear to result in the loss of detectable NF155 immunostaining at the paranodes, although the precise mechanism for this remains unknown. The partial rescue by exogenous calpain inhibition may therefore result from the partial protection of AnkyrinB. However, axonal calpains must also play a role in NF155 loss, perhaps due to the disruption of axonal anchoring proteins such as Caspr, which we previously showed was lost following acute glial injury.^5^ This would explain the partial protection of pan‐neurofascin staining by axonal *hCAST* expression. The mechanism of axonal calpain involvement at this early timepoint is unknown as no detectable axonal calcium flux is present at acute time points, and NF‐H staining is also intact, suggesting a more subtle and complicated contribution to NF155 loss. It is possible that low intracellular calcium concentrations are sufficient to activate calpain 1 and affect axonal proteins at this early stage and that this is undetectable by TNXXL calcium imaging. The interpretation of this result is confounded by the use of pan‐neurofascin antibody as the partial protection of neurofascin staining could also be a spread of NF186 from the axonal NoR.

### 4.3 Secondary axon degeneration is prevented by axonal calpain inhibition

Following acute injury to the paranodal glial membranes we previously showed early changes to the axonal paranodal (Caspr) and nodal proteins (NF186, Nav1.6, AnkyrinG), but preservation of the major axonal structural protein NF‐H, indicating a maintenance of axonal integrity at this early timepoint.^5^ As the injury time frame progressed, we found that NF‐H immunoreactivity was eventually extinguished, indicating a loss of axon integrity. Herein, we have mapped the timeframe of neurofilament decay between the acute (4 h) and extended (20 h) timepoints. We have now tested various mechanisms that might account for this.

Firstly, to address the possibility of the axonal injury being a direct result of MAC deposition on the axon: Our *Glial* mice express GM1 ganglioside on glial cells only and, despite extensive immunofluorescent investigation, it remains possible that axonal GM1 in glial mouse could have arisen through paranodal loop shedding. However, we also target the glia in wild‐type mice using an anti‐sulfatide antibody, a glially expressed lipid, and show the same results.^5^ Similarly, MAC pores can also be shed but there is no evidence that an intact MAC pore may be shed to re‐enter adjacent membranes.^35^ Axons receive energy support via Schwann cells that shuttle lactate via monocarboxylate transporters to the axons.^36^ Change in Schwann cell metabolism or lactate shuttling can cause deficits in axonal maintenance.^37^, ^38^, ^39^, ^40^, ^41^ Addition of exogenous L‐Lactate can therefore compensate for deficits in Schwann cell metabolic support to axons.^36^ We tested this in our model, but lactate supplementation did not attenuate axonal changes.

ROS may cause axon degeneration in neuropathies.^42^ Lipid‐rich myelin has a high rate of oxidative metabolism and therefore is susceptible to the oxidative stress that produces free radicals like ROS. In GBS patients, levels of some antioxidants have been shown to be reduced in the blood^43^, ^44^ though how these changes correlate to oxidative myelin or axonal damage has not been fully explored. Injured axons in an in vitro model of axonal GBS were shown to release H_2_O_2_ from mitochondria due to calcium overload.^45^ We tested whether Schwann cell injury was resulting in bystander ROS‐driven axonal damage or was resulting in hypoxia‐induced ROS generation in the axons by inhibiting ROS with YCG063. Even at high concentrations, secondary axon loss still occurred, indicating that this was not a major pathway of axonal damage in our model at this timepoint. Other cytoplasmic factors released from injured Schwann cells, such as heat shock proteins or other alarmins may be occurring in this model and could contribute to axonal injury.^46^

Secondary axon degeneration could occur as a result of a programmed process of axon degeneration. SARM1 is an injury‐induced NADase. Deletion of the SARM1 protein has been shown to protect against axon degeneration in many peripheral neuropathy models, including chemotherapy‐induced peripheral neuropathies, diabetic neuropathies as well as central nervous system models of demyelinating disease.^47^, ^48^ However, it has had mixed effectiveness in preventing secondary axon degeneration in models of different variants of genetic demyelinating neuropathies.^49^, ^50^ We have previously shown that in our model of primary axonal injury, use of WLD^s^ mice, which also show delayed Wallerian degeneration, did not protect from axonal injury.^51^ This has also been demonstrated in *SARM1* KO mice in our primary axonal injury model (unpublished observations). Here we show that secondary axonal degeneration in our model is not prevented in *SARM1* KO mice and therefore is likely also not a programmed event.

Calpain inhibition prevents both programmed and non‐programmed axon degeneration and is therefore a final executioner for many types of axon degeneration.^52^, ^53^, ^54^, ^55^, ^56^ Calpain‐mediated injury is responsible for the primary axon degeneration seen in our AMAN models.^16^, ^17^, ^18^, ^19^ Therefore, we anticipated a role for calpain in secondary axon degeneration. Using axonal calpain inhibition, we observed a major attenuating effect on secondary axonal degeneration as assessed by NF‐H loss following extended glial injury. We, therefore, demonstrate a converging pathway in both primary and secondary axon degeneration. As calpain is a calcium‐activated protease we next sought to demonstrate the events preceding calpain activation.

### 4.4 Axonal calcium flux through nanoruptures precedes secondary axon degeneration

Following extended glial injury, we demonstrated the presence of an axonal calcium flux. This became most apparent 6 to 9 h post glial injury and demonstrates that a rise in axonal calcium precedes secondary axon degeneration in this model. Measured MNTs appeared to show rises in calcium at earlier timepoints than single axons, likely due to the terminal heminode being more vulnerable to our injury than more proximal NoR due to a weaker blood nerve barrier.^16^ The source of axonal calcium could be from intracellular calcium stores, or be extracellular, entering via calcium channels. However, we have previously noted that endogenously expressed axonal cytoplasmic CFP is lost in our extended glial injury model.^5^ Additionally, here we demonstrate that despite axonal structural integrity being protected (as judged by NF‐H staining), axonal CFP loss still occurs, not being prevented by calpain inhibition. This implies that axon membrane integrity must remain impaired in extended glial injury, allowing bi‐directional flow of molecules, with calcium entering and CFP exiting. We, therefore, found it more likely that calcium entry was via a previously proposed and demonstrated pathway: nanoruptures in the axolemma.^54^, ^57^ To investigate this in the absence of any available direct imaging methods, we used labelled dextrans to functionally demonstrate the putative nanopores in the axon membrane and determine their relative sizes, concluding they are in the range of 2 to 14 nm.^58^, ^59^ This would be compatible with the leakage of small molecules such as CFP (27 kDa) and ions, whilst preserving major structural integrity. Dextran localisation appears to be axonal but low molecular weight dextrans accumulating through MAC pores on the paranode cannot be excluded.

**FIGURE 4:**
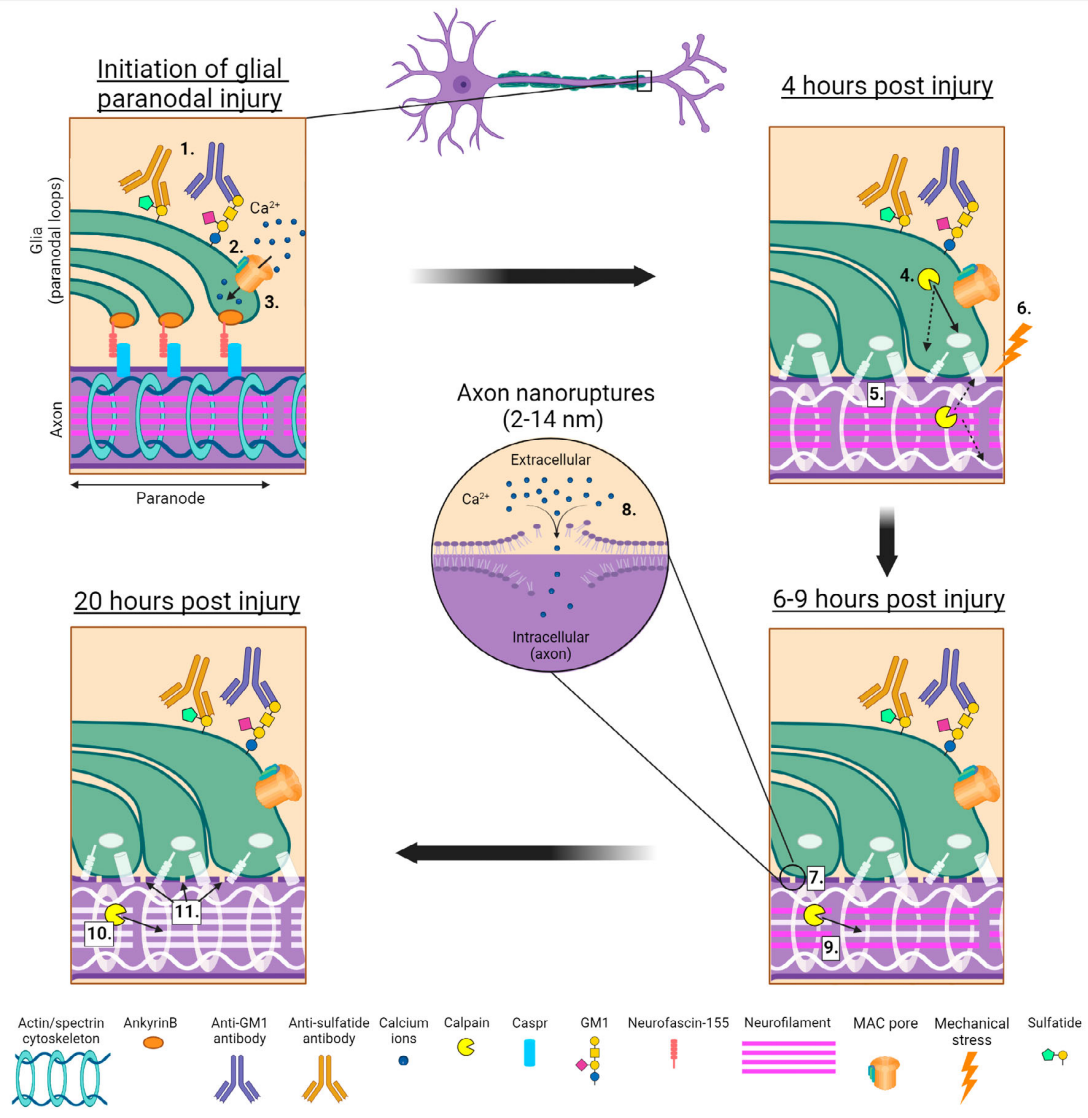
Proposed mechanism of secondary axon degeneration in mouse model of extended glial injury. 1. Antibody binds to glial paranode. 2. Complement pore is deposited in glial membrane. 3. Influx of calcium. 4. Paranodal proteins disrupted by glial calpains and membrane distortion. 5. Axon cytoskeleton is disturbed. 6. Axon undergoes mechanical stress. 7. Nanoruptures form on axon membrane. 8. Extracellular calcium enters the axon. 9. Axonal calpains activate and begin to degrade NF‐H. 10. NF‐H loss by axonal calpain is complete. 11. Nanoruptures remain present on axon membrane. Created with BioRender.com

The nanoruptures we observed are similar in size to those formed in mouse models of experimental allergic encephalomyelitis (EAE).^57^ The exact cause of the formation and appearance of these nanoruptures is unknown, but the authors speculated several potential mechanisms of rupture formation in this model, including mechanical stress, production of cytotoxic ROS or release of toxic mediators by immune cells. In our model, we have ruled out axonal metabolic insufficiency and injury due to cytotoxic ROS release from injured Schwann cells and immune cell involvement is unlikely in this ex vivo paradigm. The axon cytoskeleton, comprising periodic repeating rings of spectrins and ankyrins is critical for axonal integrity by forming a scaffolding, which also aligns to paranodal cell adhesion molecules.^60^ This cytoskeleton has a proposed role in buffering axons from mechanical stress.^61^ Therefore, we propose that the combination of distortion of the paranode and axoglial interface and disruption of axonal nodal and paranodal protein interactions causes the underlying axonal cytoskeleton to be compromised (Figure 4). With a reduced ability to buffer mechanical stress, the paranodal swelling causes compression and shearing of the axon, a known mechanism of pore formation in lipid membranes.^62^, ^63^ We speculate that the axonal paranode is a particularly vulnerable region for nanoruptures because of the tight and complex interactions between axonal and glial proteins which are necessary for maintenance of a stable node of Ranvier.^60^ The concept of nanoruptures that allows influx of extracellular calcium have been demonstrated before.^54^, ^57^ Compellingly, these studies demonstrated that calcium influx does not necessarily destine an axon for transection with ensuing destruction of the distal stump. Spontaneous membrane resealing may return an axon to its normal state. This metastable state of axon pathology infers a tipping point or threshold for calpain activation in individual axons.^54^, ^57^, ^64^ The resulting dichotomy in axonal fate may explain both why primary and secondary axon degeneration occurs to differing extents in AMAN and AIDP patients respectively.

The formation of axonal nanoruptures appears to be the driving cause of secondary axonal degeneration following selective glial membrane injury in this model system. As with primary axon degeneration, axonal calcium influx followed by the activation of calpain are the climactic events in secondary axon degeneration. Importantly, the secondary axon degeneration we have observed is not directly associated with complement cascade activation in the axolemma. The common pathway of both primary and secondary axon degeneration is, thus, at the level of calcium‐activated calpain cleavage, not complement.

We have demonstrated the central role of calpain and calpain inhibition in preventing the neurofilament breakdown occurring after extended glial membrane injury. We also observe this calpain pathway in a model of primary axonal injury, where axons directly targeted by anti‐GM1 antibody are protected by expression of axonal calpastatin and recover more rapidly.^19^ Calpain inhibition is thus a promising candidate for therapeutic intervention in primary axonal forms of GBS and may also attenuate secondary axon degeneration in demyelinating forms of GBS. As MAC pores are shed and nanoruptures reseal, the expectation would be that this shifts the metastable state in favour of axonal survival rather than transection.^54^ Calpain inhibition strategies are of potential benefit in many disorders including GBS, though the development of successful treatments has proven challenging.^65^, ^66^ In GBS, calpain inhibition as a neuroprotective strategy might be best used as a combinatorial approach along with other treatments including autoantibody depletion and complement inhibition, strategies which have been shown to be effective in both axonal and glial injury‐targeted mouse models of GBS.^27^, ^67^, ^68^

## CONFLICT OF INTEREST

The authors declare that there is no conflict of interest.

## Data Availability

The datasets analysed during the current study are available in the University of Glasgow Enlighten: Research data repository, https://doi.org/10.5525/gla.researchdata.1361.
